# Is angiotensin-converting enzyme inhibitors/angiotensin receptor blockers therapy protective against prostate cancer?

**DOI:** 10.18632/oncotarget.6837

**Published:** 2016-01-07

**Authors:** Yeqing Mao, Xin Xu, Xiao Wang, Xiangyi Zheng, Liping Xie

**Affiliations:** ^1^ Department of Urology, First Affiliated Hospital, School of Medicine, Zhejiang University, Hangzhou, China

**Keywords:** angiotensin-converting enzyme inhibitor, angiotensin receptor blocker, prostate cancer, meta-analysis, cohort

## Abstract

Emerging evidence suggests that renin-angiotensin system (RAS) may act as a molecular and therapeutic target for treating site-specific cancers, including prostate cancer. However, previous observational studies regarding the association between RAS inhibitors and prostate cancer risk have reported inconsistent results. We examined this association by performing a systematic review and meta-analysis. A total of 20,267 patients from nine cohort studies were enrolled. Compared with non-users of RAS inhibitors, individuals using RAS inhibitors had a reduced risk of prostate cancer (RR 0.92, 95 % CI 0.87-0.98), without statistically significant heterogeneity among studies (*P* = 0.118 for heterogeneity, I^2^ = 37.6 %). In addition, when subgroup analyses by study quality and number of cases, more statistically significant associations were observed in studies of high quality (RR 0.93, 95 % CI 0.88-0.97) and large sample size (RR 0.94, 95 % CI 0.91-0.98). There was no evidence of significant publication bias with Begg's test (*P* = 0.602) or with Egger's test (*P* = 0.350). Overall, this study indicates that use of RAS inhibitors may be associated with a decreased risk of prostate cancer. Large-scale well designed studies are needed to further explore this association.

## INTRODUCTION

Prostate cancer is the second most commonly diagnosed cancer in men worldwide, with 1,111,700 new cases and 307,500 deaths estimated to have occurred in 2012 [1]. The most well-established risk factors for prostate cancer are age, positive family history, and race/ethnicity [2]. Less well-established but modifiable risk factors include unhealthy behaviors (e.g., lack of physical activity [3]) and eating fewer vegetables, such as carrots [4] and cruciferous vegetables [5]. In addition, it has been proposed that statins [6], aspirin [7], and metformin [8] have additional beneficial anti-carcinogenic effects and are linked to a lower risk of prostate cancer, although the evidence has been conflicting.

Renin-angiotensin system (RAS) inhibitors, such as angiotensin-converting enzyme inhibitors (ACEIs) and angiotensin II receptor blockers (ARBs), are commonly used in the treatment of hypertension across the world. Beyond cardiovascular effects, there is some evidence that RAS inhibitors might reduce risk of site-specific cancers (e.g., esophageal [9] and colorectal cancer [10]) and improve clinical outcomes of cancer patients combined or not combined with chemotherapy/radiotherapy [11-14]. A potential mechanism underlying this phenomenon is that RAS signaling is able to stimulate cell proliferation in human cancers by directly affecting tumor and stromal cells and by indirectly regulating the growth of vascular cells during angiogenesis [15]. Therefore, RAS may act as a molecular target in cancer therapy.

*In vitro* and *in vivo* studies of prostate cancer, a growing body of evidence has indicated that drugs targeting the RAS could inhibit tumor growth and promote apoptosis, thus may open up new therapy options for prostate cancer patients [16]. However, the findings from epidemiological studies on the association between use of RAS inhibitors and prostate cancer risk are not completely consistent [17-20]. Considering the potential huge value of RAS inhibitors for prostate cancer prevention and treatment, we performed this meta-analysis to summarize and to quantity the existing evidence on the relationship between RAS inhibitors and prostate cancer based on all relevant cohort studies.

## RESULTS

### Literature search and study characteristics

**Figure 1:**
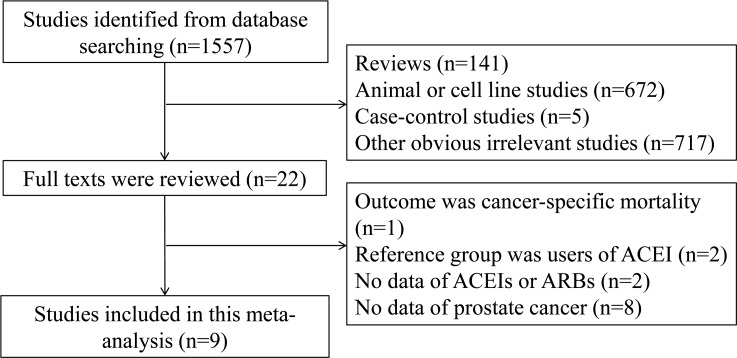
Process of study selection

The detailed steps of our literature search are presented in Figure 1. Nine eligible studies [17-25] were eventually included in this meta-analysis of the association between use of RAS inhibitors and prostate cancer risk. These studies (six cohort and three nested case-control studies) were conducted in the following geographical regions: North America (*n* = 4), Europe (*n* = 4), and Asia (*n* = 1). All of the included studies were published between 2001 and 2013, including a total of 20,267 cases. Information on exposure (RAS inhibitors) and outcome (prostate cancer) was mainly obtained by medical records. Four studies used hazard ratio (HR), two used RR, two used odds ratio (OR), and one used standardized incidence ratio (SIR). The study quality scores, assessed by the NOS, ranged from 5 to 8 (with a mean of 7). Table 1 shows the characteristics of each study included in this meta-analysis.

**Table 1:** Characteristics of the studies included in meta-analysis of association between use of RAS inhibitors and prostate cancer risk

Study	Cohort information (population)	Exposure assessment	Outcome assessment	Study design, cases/cohort (controls)	Age, yr, mean (range)	Follow-up, yr, mean (range)	Type of medication (reference group)	Matched or adjusted factors	NOS score
Fitzpatrick et al., 2001, USA	Men with or without hypertension (subgroup of Cardiovascular Health Study)	Medication inventory of drugs	Medical records and self-report	Cohort, 209/2442	73.3 (≥ 65)	5.6 (NA)	ACEIs (no antihypertensive drug)	Age, race, BMI	6
Friis et al., 2001, Denmark	Hypertensive patients receiving drug treatment (Prescription Database of North Jutland County)	Prescription database	Cancer registries	Cohort, 60/8865	62 (NA)	3.7 (0–8)	ACEIs (general population)	NA	5
Ronquist et al., 2004, UK	Review of data in GPRD (1995-1999) for cohort of men aged 50–79 yr	Computerized medical records	Computerized information	Nested case-control, 1013/10000	NA (50–79)	NA	ACEIs (no ACEIs)	Age, calendar year, prostatism, other antihypertensive medication usage	7
van der Knaap et al., 2008, Netherlands	Individuals with or without hypertension (subgroup of Rotterdam study)	Pharmacies	Medical records	Cohort, 199/7679	70.4 (≥ 55)	9.6 (NA)	ACEIs or ARBs (no ACEIs or ARBs)	Age, BMI, use of salicylates, DM, hypertension, MI	8
Assimes et al., 2008, Canada	Saskatchewan Heath database for cohort of current users of antihypertensive drugs between 1980 and 1987	Prescription database	Cancer registries	Nested case-control, 975/9583	71.8 (NA)	NA	ACEIs or ARBs (diuretic)	Age, all measured comorbid conditions, exposure to all other classes of antihypertensive medication	7
Rodriguez et al., 2009, USA	Men with or without hypertension (subgroup of CPS II Nutrition Cohort)	Questionnaires	Medical records and cancer registries	Cohort, 3031/48389	NA (50–74)	6.32 (NA)	ACEIs (no use of antihypertensive drug)	Age, race, BMI, education, family history of prostate cancer, history of DM, history of PSA screening, history of heart disease or bypass surgery, use of cholesterol-lowering drugs, concomitant use of other anti-hypertensive drugs.	8
Azoulay et al., 2012, UK	Patients who prescribed an antihypertensive agent (GPRD: 1995-2008)	Medical records in GPRD	Medical records in GPRD	Nested case-control, 5734/58763	63.4 (NA)	6.4 (2-16)	ACEIs or ARBs (diuretic and/or beta-blocker)	Age, calendar year of cohort entry, prevalent user status, duration of follow-up. alcohol, smoking, BMI, hypertension, CHF, CHD, DM, previous cancer, the ever use of aspirin, other NSAIDs, statins, BPH, prostatitis, use of 5-ARIs	8
Wang et al., 2013, China	Subjects exposed to ARBs ≥180 days (Taiwan NHIRD: 1997-2009)	Medical records in NHIRD	Medical records in NHIRD	Cohort, 271/43478	62 (NA)	4.8 (≥ 2)	ARBs (no ARBs)	Age, comorbidities, calendar year of cohort entry	7
Rao et al., 2013, USA	Veterans who were classified into either ARB treated or not-treated in 1:15 ratio	Medical records	Cancer registries	Cohort, 8775/543824	63.6 (55-74)	NA (≤ 8)	ARBs (no ARBs)	Intention-to-treat inverse-probability-of-treatment-weighted was used to balance differences between the groups	7

ACEIs, angiotensin-converting-enzyme inhibitors; ARBs, angiotensin-receptor blockers; yr, year; NOS, Newcastle-Ottawa Scale; BMI, body mass index; GPRD, General Practice Research Database; DM, diabetes mellitus; MI, myocardial infarction; CPS II, Cancer Prevention Study II; CHF, congestive heart failure; CHD, coronary heart disease; NSAIDs, nonsteroidal anti-inflammatory drugs; BPH, benign prostatic hyperplasia; 5-ARIs, 5-alpha reductase inhibitors; NHIRD, National Health Insurance Research Database

### Overall and subgroup analysis

**Figure 2:**
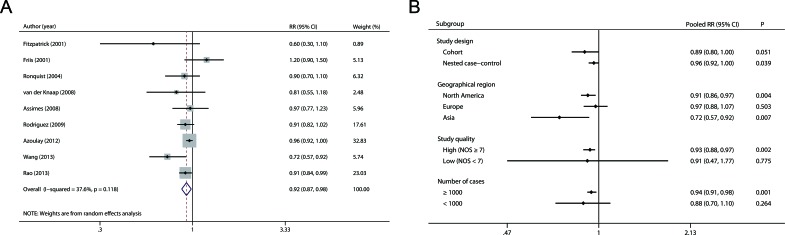
Overall **A.** and subgroup **B.** analyses of the association between use of RAS inhibitors and prostate cancer risk.

The multivariable-adjusted RRs for each study and for the combination of all included studies are shown in Figure 2A. A significant decrease in the risk of prostate cancer (RR 0.92, 95 % CI 0.87-0.98, *P* = 0.012) was observed among individuals using RAS inhibitors. There was moderate but not statistically significant heterogeneity among studies (*P* = 0.118 for heterogeneity, I^2^ = 37.6 %).

Next, we carried out subgroup analyses by study design, geographical region, study quality, and number of cases (Figure 2B and Supplement Table S1). When stratified by study design, the RRs (95 % CI) were 0.89 (0.80-1.00) and 0.96 (0.92-1.00) for cohort and nested case-control studies, respectively. In the subgroup analyses separated by geographical region, more pronounced associations were detected in studies from North America (RR 0.91, 95 % CI 0.86-0.97) and Asia (RR 0.72, 95 % CI 0.57-0.92) compared with studies from Europe (RR 0.97, 95 % CI 0.88-1.07). In addition, when stratifying by study quality and number of cases, statistically significant associations were observed in studies with high quality (RR 0.93, 95 % CI 0.88-0.97) and large sample size (RR 0.94, 95 % CI 0.91-0.98) but not in studies with low quality (RR 0.91, 95 % CI 0.47-1.77) or small sample size (RR 0.88, 95 % CI 0.70-1.10).

### Evaluation of heterogeneity

**Figure 3:**
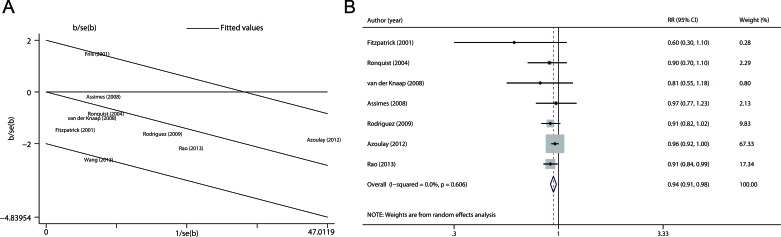
Heterogeneity analyses **A.** Galbraith plot analysis was performed to evaluate possible sources of heterogeneity. **B.** Summary risk estimates and 95% CIs for use of RAS inhibitors and prostate cancer risk after removing the studies that contributed to the heterogeneity.

We used the Q statistic and the I^2^ index to assess heterogeneity in this meta-analysis. As shown in Figure 2A, moderate heterogeneity was observed among the studies (*P* = 0.118 for heterogeneity, I^2^ = 37.6 %). Then we performed Galbraith plot analysis and found that studies by Friis et al. [21] and Wang et al. [25] were the possible sources of heterogeneity (Figure 3A). After removing these two studies, there was no study heterogeneity (*P* = 0.606, I^2^ = 0.0 %) and the combined RR remained statistically significant (Figure 3B, RR 0.94, 95 % CI 0.91-0.98).

### Sensitivity analysis

**Figure 4:**
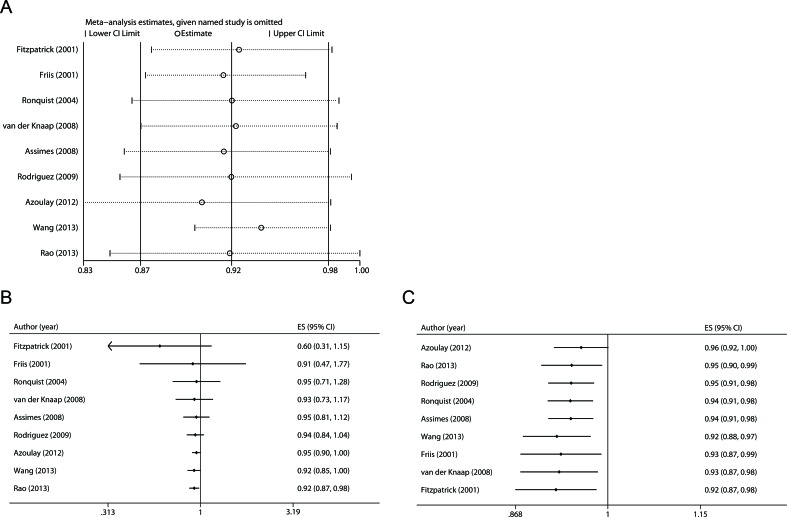
Sensitivity and cumulative meta-analyses **A.** Sensitivity analysis was carried out whereby each study was removed in turn and the pooled estimate was recalculated. **B.** Cumulative meta-analysis was performed according to date of publication. **C.** Cumulative meta-analysis was performed by sorting the studies from most precise to least precise.

We first examined the influence of each study on the combined RR by repeating the meta-analysis after omitting each study in turn. The nine study-specific RRs are shown in Figure 4A. Except the omission of the study by Rao et al. [17], which led to a borderline significant estimate (RR 0.92, 95 % CI 0.84-1.00), the other eight pooled RRs remained statistically significant. In addition, considering studies using SIR may underestimate the true risk when use of RAS inhibitors in the general population is high, we removed the study by Friis et al. [21] and re-estimated the pooled RR, which was 0.92 (0.87-0.97).

### Cumulative meta-analysis

Cumulative meta-analysis was carried out by repeatedly re-running the meta-analysis each time adding a new study. We first performed the cumulative meta-analysis according to date of publication. As shown in Figure 4B, the pooled RR became statistically significant when the study by Rao et al. [17] completed in 2013 was added. Then we conducted the cumulative meta-analysis by sorting the studies from most precise to least precise to assess the small-study effect. As shown in Figure 4C, the cumulative effect was not changed when smaller studies were added.

### Publication bias

**Figure 5:**
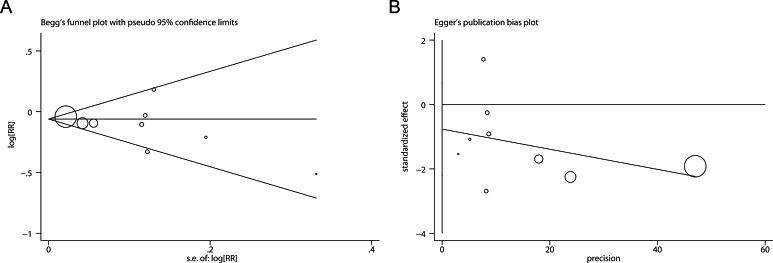
Publication bias analyses **A.** Begg's test (rank correlation method). **B.** Egger's test (linear regression method).

There was no evidence of significant publication bias with Begg's test (Figure 5A, *P* = 0.602) or with Egger's test (Figure 5B, *P* = 0.350).

## DISCUSSION

The current meta-analysis summarized the results of observational studies on the association between use of RAS inhibitors and prostate cancer risk, including six cohort studies and three nested case-control studies. The results indicated that individuals treated with RAS inhibitors had a significant reduced risk of prostate cancer.

Recently, the potential relationship between use of RAS inhibitors and cancer risk has captured attention and published results on this association have been contradictory. An early meta-analysis performed by Sipahi et al. [26] included five clinical trials and suggested that ARBs was associated with a modestly increased risk of new cancer occurrence. Another meta-analysis of randomized controlled trials by Bangalore et al. [27] also concluded increased risk of cancer with the combination of ACEI and ARBs could not be ruled out. However, recently Connolly et al. [28] summarized the evidence from fifteen clinical trials and found there was no significant increase in the total or site-specific cancer risk from use of ARBs. Sipahi et al. [29] completed a new meta-analysis of fourteen trials and reported ACEI was not significantly related with occurrence or death of cancer. Although clinical trials included in prior meta-analyses had strong internal validity, these meta-analyses also had several methodological limitations. Cancer was not the primary outcome of interest and duration of follow-up for cancer detection in most of the trials was short, ranging from one to five years.

On the other hand, emerging observational studies have indicated a decrease risk with the use of RAS inhibitors in various cancers, although conflicts still exist [30, 31]. Use of RAS inhibitor or combination with conventional chemotherapy/radiotherapy also has been shown to be associated with better clinical outcome [11, 13, 32]. For site-specific cancer risk, a recent published meta-analysis reported that individuals using RAS inhibitors were associated with a decreased risk of colorectal cancer based on eleven observational studies [10]. To the best of our knowledge, there is no meta-analysis that has summarized the evidence on the association between RAS inhibitors and prostate cancer risk.

Although moderate heterogeneity was detected among the included studies, Galbraith plot analysis implied our findings were less vulnerable to the effects of heterogeneity. Furthermore, cumulative meta-analysis based the precision of included studies, Begg's test, and Egger's test indicated no obvious evidence of small-study bias. The combined RRs in sensitivity analyses were stable and not substantially influenced by a specific study. In subgroup analyses by study quality and sample size, more pronounced associations were observed in studies with high quality and large sample size. All of these results confirmed the reliability and robustness of our findings.

An inverse association between use of RAS inhibitors and risk of prostate cancer is biologically plausible. Some studies [33, 34], as well as our previous study [35], support the hypothesis that ACE genetic variants may affect the production of angiotensin II (Ang II) and the progression of human prostate cancer. Previous studies have confirmed that Ang II can promote angiogenesis, an important determinant in tumor growth and spread [36]. It has also been indicated that Ang II promotes the secretion of several growth factors and cytokines [37]. In addition, activation of angiotensin-II type 1 receptor (AT1R) in LNCaP, DU145, and PrSC cells resulted in increased MAPK activation, JAK-STAT signaling, and cell proliferation [37, 38]. ARBs, including telmisartan and candesartan, have been reported to inhibit AT1-R expression, suppress cell proliferation, and augment apoptosis in prostate cancer [39-41]. ARBs were also able to inhibit MCP-1 expression and blocks macrophage infiltration in castration-resistant prostate cancer [42]. All these data support a new concept that RAS inhibitors are promising potential chemopreventive and chemotherapeutic agents for prostate cancer.

There are several important limitations that need to be considered in interpreting the findings of this meta-analysis. First, the quality of meta-analysis depended on the included studies, which may fail to fully adjust for the confounding factors. A meta-analysis is unable to solve this problem and inadequate control of all known confounders may bias the risk estimate. Second, the current evidence on this association remains limited. Only nine studies were included in this meta-analysis and the pooled RR just reached statistical significance when the most recent study by Rao et al. conducted in 2013 was added. Lastly, people treated with RAS inhibitors are under increased medical surveillance, which may lead to detection bias. In addition, they may be more likely to change their unhealthy lifestyle, which may also confound the “true” relationship.

## CONCLUSION

This meta-analysis indicates that use of RAS inhibitors may be associated with a decreased risk of prostate cancer. Considering the pooled RR is approaching 1, this evidence should be interpreted with caution although the *P* value is statistically significant. Large-scale well designed studies are needed to further explore this association.

## MATERIALS AND METHODS

### Publication search

We performed a literature search of published studies in July 2015 using PubMed, Web of Science, and the Chinese National Knowledge Infrastructure (CNKI) databases. Considering some studies that assessed the relationship between RAS inhibitors and overall cancer risk also reported site-specific cancer risk, we broadened the search by using a loose search algorithm (see Supplementary Material for the detailed search strategies). We also checked the reference lists from retrieved articles and related reviews to identify any additional relevant studies. There were no limitations on language or publication date. This systematic review was designed, conducted, and reported according to the standards of quality for reporting meta-analyses [43].

### Study selection

The studies included in this meta-analysis met all of the following criteria: (*i*) the exposure of interest was RAS inhibitors; (*ii*) the outcome of interest was prostate cancer; (*iii*) study design was cohort or nested case-control; and (*iv*) the effect size estimates with their corresponding 95 % confidence intervals (CIs) were provided (or relevant data were available to calculate these values).

### Quality assessment

Two authors (Y.Q.M and X.X.) evaluated the quality of each study by using the Newcastle-Ottawa Scale (NOS) (http://www.ohri.ca/programs/clinical_epidemiology/oxford.asp). NOS is an eight-item instrument that allows for the assessment of selection (four items), comparability (one item), and outcome (three items). Each item corresponds to one point, except for comparability (two points). Thus the range of possible scores is 0-9 points. Studies are assigned as high quality if the score is 7-9 points.

### Data extraction

The following data were extracted from each study: first author's name, publication year, the country where the study population came from, study design, age of study population, sample size, method of exposure and outcome assessment, duration of follow-up, adjusted effect size estimates with their corresponding 95 % CIs, and matched or adjusted confounders in the design or data analysis. Data were collected independently by two authors (X.X. and Y.Q.M) using a predefined data collection form. Discrepancies were resolved by consensus or consulting a third reviewer.

### Statistical methods

Relative risks (RRs) and their 95 % CIs were used to calculate and assess the strength of the association between use of RAS inhibitors and prostate cancer risk. A random-effects model proposed by DerSimonian and Laird [44], which takes into account both within- and between-studies variability, was adopted to estimate the pooled RR and its 95 % CI. Subgroup analyses were carried out by study design, geographical region, study quality, and number of cases.

The heterogeneity of RRs across the studies was tested by the Cochran Q and the I^2^ index [45]. The level of significance for Cochrane Q was set at 0.1. The value of I^2^ was used to quantify the impact of heterogeneity (I^2^ < 25 %: no heterogeneity; I^2^ = 25-50 %: moderate heterogeneity; I^2^ > 50 %: large or extreme heterogeneity). Furthermore, the Galbraith plot [46] was used to investigate possible sources of heterogeneity, and a re-analysis of the data was carried out after exclusion of the studies thought to be sources of heterogeneity.

Sensitivity analysis was performed whereby each study was removed in turn and the pooled estimate was recalculated to determine the influence of each study. Cumulative meta-analysis was also carried out by sorting the studies based on publication year or from most precise to least precise.

Potential publication bias was assessed using Begg's test (rank correlation method) [47] and Egger's test (linear regression method) [48]. All of the statistical analyses were performed with STATA 11.0 (StataCorp, College Station, TX), using two-sided *P* values (except where otherwise specified, the level of significance was set at 0.05).

## SUPPLEMENTARY MATERIAL TABLE


