# Supplementary material for: Is angiotensin-converting enzyme inhibitors/angiotensin receptor blockers therapy protective against prostate cancer?
Source: Oncotarget. 2016 Jan 7;7(6):6765–73. doi: 10.18632/oncotarget.6837 (PMC4872747; doi:10.18632/oncotarget.6837)
Supplement: Supplementary file 1 [file oncotarget-07-6765-s001.pdf]

Is angiotensin-converting enzyme inhibitors/angiotensin receptor blockers therapy protective against prostate cancer?

Supplementary Material

Table S1. Subgroup analyses of the association between use of RAS inhibitors and prostate cancer risk

Subgroup	Included studies	No. of cases	Pooled RR (95 % CI)	*P*	Heterogeneity		
					Q	I^2 (%)	*P*
**Total**	9	20,267	0.92 (0.87-0.98)	0.012	12.82	37.6	0.118
**Study design**							
Cohort	6	12,545	0.89 (0.80-1.00)	0.051	10.08	50.4	0.073
Nested case-control	3	7,722	0.96 (0.92-1.00)	0.039	0.31	0.0	0.855
**Geographical region**							
North America	4	12,990	0.91 (0.86-0.97)	0.004	1.86	0.0	0.601
Europe	4	7,006	0.97 (0.88-1.07)	0.503	4.00	25.0	0.261
Asia	1	271	0.72 (0.57-0.92)	0.007	NA	NA	NA
**Study quality**							
High (NOS ≥ 7)	7	19,998	0.93 (0.88-0.97)	0.002	7.47	19.7	0.279
Low (NOS < 7)	2	269	0.91 (0.47-1.77)	0.775	3.79	73.6	0.052
**No. of cases**							
≥ 1000	4	18,553	0.94 (0.91-0.98)	0.001	2.00	0.0	0.573
< 1000	5	1,714	0.88 (0.70-1.10)	0.264	10.39	61.5	0.034

RAS, renin-angiotensin system; No., number; RR, relative risk; CI, confidence interval; NOS, Newcastle-Ottawa Scale; NA, not available.

Search algorithm

(“cancer” OR “carcinoma” OR “neoplasm” OR “tumor” OR “malignancy”) AND (“angiotensin-converting enzyme inhibitor*” OR “angiotensin converting enzyme inhibitor*” OR “angiotensin I-converting enzyme inhibitor*” OR “angiotensin I converting enzyme inhibitor*” OR “ACE inhibitor*” OR “ACEI” or “captopril” OR “ramipril” OR “cilazapril” OR “enalapril” OR “fosinopril” OR “perindopril” OR “imidapril” OR “lisinopril” OR “moexipril” OR “quinapril” OR “trandolapril” OR “angiotensin receptor blockade” OR “angiotensin-receptor blockade” OR “angiotensin receptor blocker*” OR “angiotensin-receptor blocker*” OR “angiotensin II receptor blocker*” OR “angiotensin-II receptor blocker*” OR “angiotensin receptor antagonist” OR “angiotensin-receptor antagonist” OR “angiotensin II receptor antagonist*” OR “angiotensin-II receptor antagonist*” OR “angiotensin II antagonist*” OR “angiotensin-II antagonist*” OR “AT1 receptor antagonist*” OR “AT1-receptor antagonist*” OR “ARB” OR “ARBs” OR “sartan*” OR “irbesartan” OR “eprosartan” OR “losartan” OR “telmisartan” OR “valsartan” OR “olmesartan” OR “candesartan” OR “renin-angiotensin system inhibitor*” OR “renin angiotensin system inhibitor*” OR “renin-angiotensin-aldosterone system inhibitor*” OR “renin angiotensin aldosterone system inhibitor*” OR “RAS inhibitor*”)
